# The Gendering of Infectious Disease: Classifying Male and Female Causes of Death in the Netherlands and Norway, 1880–1910

**DOI:** 10.1093/shm/hkab084

**Published:** 2021-08-25

**Authors:** Hilde L Sommerseth, Evelien C Walhout

**Affiliations:** Leiden University, Department of History, Doelensteeg 16, 2311 VL Leiden, The Netherlands

**Keywords:** mortality, cause of death, airborne infectious diseases, sex differentials, gender roles

## Abstract

This article explores sex and gender patterns in mortality, based on individual-level causes of death (CODs) in two urban communities, obtained from civil and parish registers. By analysing CODs for the period 1880–1910 for Roosendaal (Netherlands) and Trondheim (Norway) we investigate how notions of sex and gender were reflected in cause-specific mortality rates for adults and in the registration of CODs by local authorities. Our findings show (i) excess male mortality among age group 15–70, (ii) airborne infectious diseases were responsible for most deaths in both cities, but did not show a distinct gender pattern, (iii) TB appeared to be more location-specific than gender-specific. However, the level of variation and specification in TB COD terms was higher among women in both locations.

This article explores trends of cause-specific mortality for men and women as well as differences in the way their causes of death (hereafter CODs) were reported. The data are obtained from parish and civil registers of two late nineteenth-century urban communities. Whereas most studies of trends in cause-specific mortality focus on aggregated data we provide analyses based on individual-level CODs reported in the period 1880–1910 for the cities of Roosendaal in the Netherlands and Trondheim in Norway. In addition to sex differentials we are particularly interested in how notions of gender might have affected conceptualizations of disease and how this was reflected in the reporting and classification of CODs by contemporary authorities in the two cities. As an in-depth case study, we focus on sex-differentiated patterns in airborne infectious diseases among adolescents and adults because these patterns are affected by both biological and behavioural factors. We will compare the patterns in age and sex-specific mortality we observe in the two locations to see if there are differences. We will also examine cause-specific mortality rates in our two populations to see whether there are differences by sex, particularly in the infectious diseases, which were still important killers in the late nineteenth century. Furthermore, we will consider the terms in which CODs were recorded, and whether there were differences according to the sex of the deceased. We will then offer explanations of the differences we observe.

Previous research has shown that the way individual CODs were recorded and categorised by local and national authorities varied significantly over time and place.^1^ Such variations might arise as a result of the identity or character of the individuals responsible for reporting the death; through differences in the likelihood that a dying patient would be seen by a medical expert before their death; because of varying levels of—or changes in—medical knowledge; or through disparities in terminology over time or across space. In addition to these findings, this article aims to discuss the extent to which variations in how mortality and CODs were reported were affected by contemporary notions on, conceptions of, and medical discourse regarding gender, disease, and death. In the past, just as today, concepts of gender and disease were socially constructed and then, as now, these constructs had a significant influence on medical statistics, which are therefore a reflection of how a society understands and classifies illness. That understanding and the classification processes used can determine who in a particular society is considered to be sick and eligible for treatment of some kind.

Sex differences in disease patterns and the current mortality gap between the genders in Western countries have attracted a lot of attention. The gap in longevity between the genders, with females living longer than males, has been explained by a combination of biological and social factors. Biologists consider men and women to be constitutionally and genetically different. Social scientists argue that the differentials are externally driven, using the concept of gender to hypothesise that males and females and the way they are perceived differ socially, culturally and behaviourally and that these differences have both direct and indirect consequences for health and mortality outcomes. The biological concept of ‘sex’ and the socio-cultural concept of ‘gender’ are thus each considered to have an important impact on health, although they may also act interdependently.^2^

When mortality is considered using the biological category ‘sex’, men are seen to experience excess mortality from almost all types of disease.^3^ Biological factors are most significant in early life: baby girls are less likely to suffer from congenital abnormalities and are less vulnerable in the days immediately after birth.^4^ Adult women generally cope and adjust better to stress and accidents and are less susceptible to infectious disease. Sex-specific disease and mortality patterns are also affected by genetic and immunological factors which generally favour women, even under epidemic conditions.^5^ Consequently, we expect the biological factor ‘sex’ to influence differentials observed in historic COD-patterns.

We also hypothesise that ‘gender’ affected health and mortality. Gender roles—the traits and expectations societal norms ascribe to males and females—are related to lifestyle, occupation, risk-taking and other types of behaviour.^6^ Such social and behavioural characteristics are key factors influencing differences in rates of mortality between the sexes.^7^ Historically, for example, higher rates of smoking and alcohol use have been attributed to male socialization practices.^8^ Likewise, women’s reproductive role meant that in past societies men were expected to undertake more dangerous tasks, and this exposed them to greater risks of mortality. Nevertheless, pregnancy and childbirth put women in the past at substantial risk of maternal mortality.^9^

Historical research has also focused on excess mortality amongst young girls in the past. This too can be explained by both biological and socioeconomic and cultural factors.^10^ Tuberculosis (TB), a major killer in the past, targeted women in their teenage years and early in their childbearing period.^11^ Although the sex differential in TB mortality may have been due in part to biological mechanisms, women’s vulnerability to the disease was enhanced by the expectation that they would act as caregivers and because the social and economic position of girls within poor families was particularly precarious.^12^ It has been stated that female TB mortality rates were higher in areas where dwelling quality was poor and overcrowding was an issue: women and children spent relatively more time in such dwellings than their menfolk, who spent their long working-days elsewhere.^13^ Recent research did not find strong evidence for the ‘bargaining-nutrition’ arguments and concluded that behavioural factors, particularly related to the working conditions females had to endure in overcrowded factories, were more likely to be the cause of female TB mortality rates being higher than male.^14^

Another mechanism through which gender plays a role in sex-differentiated mortality concerns the framing of diseases as either male or female. This process constructs an illness as particularly ‘male’ or ‘female’ depending on the symptoms and incidence of the disease, as well as the gender of the patient. Cancer, hysteria and melancholia are examples of diseases that were framed as ‘female’.^15^ Assumptions about diseases and gender could have unfortunate consequences for suffering patients. For example, in modern society heart attacks tend to be more associated with men, and as a result women can be less aware that they too are at risk. In the same way, the binary concept which holds that the X-chromosome is ‘female’ and the Y-chromosome ‘male’, has shaped modern theories and models of medical research. To analyse how such concepts of ‘sexed’ bodies and ‘gendered’ diseases manifested themselves in historical medical discourse, feminist scholars have created a large body of literature, mostly based on qualitative sources such as medical drawings and textbooks.^16^ We assume that the historical practices associated with the diagnosis and classification of CODs were also conducted through gendered lenses. Medical statistics can therefore be seen as reflecting not only a society’s socio-cultural notions of disease but also their attitudes to gender.

Besides studying biological differences in male and female COD-patterns, this article argues that historical medical practice was indeed shaped by local notions concerning gender and that this can be seen in the ways that particular societies conceptualized disease and death and reported and classified CODs. Despite progress in medical knowledge over time, diagnostic practices applied when dealing with men and women continued to differ, and still do today.

This study offers an evaluation of the comparability of CODs in two localities, through an examination of local recording practices and a consideration of the gender profiles of particular individual CODs within the two distinct disease environments. We selected our two study locations because they both had individual-level data on CODs, but principally because of the sharp contrast in their socio-economic outlooks. Both communities were located in Northwest Europe, and therefore might be expected to have shared similar attitudes to gender, with similar levels of gender equality and female access to the labour market, as well as having similar demographic systems with comparable age at marriage and levels of reproduction. We should remember, however, that such socio-economic and demographic contexts can differ extensively from one region to another. We consider our findings, and the explanations for them, in the context of the local medical infrastructure and socio-economic, environmental and epidemiological conditions in our two study communities.

This article is formed of six sections. Following this introduction, the section ‘The Populations of Roosendaal and Trondheim’ describes the populations of the two cities we have chosen to study: Roosendaal and Trondheim. The section ‘Sources’ discusses the sources and the section ‘Causes of Death Data’ reflects on the coding and classification procedures. The section ‘Findings’ presents the results of our analysis. We followed a four-step approach in order to examine our main research questions. The first part of the section ‘Findings’ focuses on overall age- and sex-specific mortality rates and focuses on the question: were there differences in the mortality patterns of men and women in these two locations? The second part of the section examines sex-specific patterns in mortality from broad categories of COD amongst adolescents and adults. It also deals with the question of whether or not the gender roles of the two sexes, particularly in regard to their differential participation in the labour market, were responsible for the differences observed in COD patterns by sex and location. The third part of the section ‘Findings’ reflects on differences seen between the sexes in mortality rates from the main COD category in both our populations: airborne infectious diseases and, more specifically, from two specific CODs within this category: TB and pneumonia. The final part of the section ‘Findings’ presents an in-depth analysis of the individual-level CODs recorded in the death and burial registers and asks whether the reporting of the specific CODs is equally detailed for both sexes and whether differences in reporting practices can be seen between Roosendaal and Trondheim. Section 6 brings the paper to a close with a short discussion of our findings.

## The Populations of Roosendaal and Trondheim

### Roosendaal

Roosendaal is a small town located in the southern part of the Netherlands, situated in the predominantly Catholic Brabant region. It started growing around 1850, thanks to various industrial and infrastructural developments such as the construction of a new railway, connecting Roosendaal to the port cities of Rotterdam and Antwerp.^17^ Towards the end of the nineteenth century it experienced rapid population growth, the number of its inhabitants growing from about 8,800 in 1880 to more than 16,700 in 1910.

As in most Dutch towns around 1900, reports from public health officials reveal complaints about the lack of attention to hygiene in public spaces. Piles of garbage and human waste were left to fester, the streets had no proper drainage, and there were pools of stagnant water which were seen as a major threat to public health. Even although the public health officers advised the local authorities to take action on these matters, little seems to have been done; there was no proper sewage system installed until the 1930s, for instance.^18^ The housing conditions in the town were also poor and this led to low standards of personal hygiene. In the slums, where the very poorest lived, several families had to share a toilet.^19^ In the Netherlands local officials were supposed to inspect the quality of each town’s food and water supplies, but in Roosendaal they rarely did. The quality of milk, usually supplied unpasteurised from local farmers, was poor; when inspected it often proved to be contaminated with farmyard manure. Untreated milk was a source of infections which led to abdominal tuberculosis and intestinal conditions but, nevertheless, local authorities failed to take the measures necessary to remedy the situation.^20^ The lack of intervention meant that infectious diseases posed a constant threat to the health of the town’s inhabitants.

Life in Roosendaal gradually underwent a transformation after 1850 as increasing numbers of the population started to work in the town’s many small-scale factories. Nevertheless, even by the 1890s only a third of the male population were employed in the factories, which mostly processed agricultural products, and another third still worked in agriculture. After 1860 a growing proportion of the male labour force was employed in construction of public infrastructure.

Women in Roosendaal were active in the local economy and as the town underwent its modest industrialization, women’s contribution increased even more as the factories presented them with the opportunity to work outside the home. However, the factories also offered more dangerous, unsafe and insanitary working conditions; the machinery was often hazardous, regulation insufficient and the workers endured extremely high temperatures on the shop floor.^21^

Women were also active elsewhere in the local labour market. The censuses of 1889 and 1899 show women working in agriculture, sewing, embroidering and laundering, and as shopkeepers or the managers of small family businesses. In addition, many young women worked as domestic servants or as milk maids on local farms.

For most women in Roosendaal marriage and childbirth brought change to their participation in the labour force. Contemporaries repeatedly expressed concerns over the fact that married women were working in the town’s factories. The local Catholic Church viewed women’s factory work as problematic and strongly opposed the participation of girls and women in the labour force in general. The Church’s main concern was that women were working alongside men on the factory floor, regardless of their marital status. Towards the end of the nineteenth century, there was greater regulation of women’s work, particularly if it involved major risks to health. Subsequently, laws regulating working hours were introduced and legislation was passed which meant that female factory workers who had given birth had to take unpaid time off work to recover.

From the 1870s labour restrictions also applied to children. Boys and girls were a source of cheap labour and were particularly sought after for jobs where their small size was an advantage. Although children under the age of 12 were banned from participating in the workforce in 1874, in practice, many children continued to be employed. Young girls, like older women, earned far less than their male counterparts.

### Trondheim

Trondheim, Norway’s third largest city, is located on the shores of a fjord. Traditionally, it was the administrative centre for the north of Norway. The population increased from 22,800 inhabitants in 1875 to 46,200 in 1910.^22^ Compared to other Norwegian cities, Trondheim’s population grew more rapidly than that of Bergen, but more slowly than that of Kristiania, the capital. The growth was due to both a surplus of births over deaths and in-migration.

The early nineteenth century saw great improvements in Trondheim’s public health infrastructure. By the 1850s the city had a system delivering clean water to the inhabitants, and in the 1880s this was extended to deliver clean water directly into people’s homes. City development in this period led to rising social inequality in water provision, however. Improvements in personal hygiene were a top priority for the local Health Commission and around 1900, two public bathhouses were built. Generally speaking, lower working class families were predominantly clustered in the eastern part of the city where they lived in small, damp houses without water closets. The city council received repeated complaints about the ‘stink’ that arose from farmers emptying the city’s toilet pails and transporting the excrement to their fields using open farm carts.^23^

The labour market in Trondheim was more varied than that in Roosendaal. Around 1800, the city’s men were employed in all sorts of industries: fishing, timber production, copper mining, trade, shipping and shipbuilding. Occupations related to the sea formed the basis of Trondheim’s economy, and the harbour was the most vibrant area of the city. During the nineteenth century, Trondheim became the economic centre of the surrounding region, and most goods leaving and entering the hinterland had to pass through the city, which had international connections by sea and rail to a variety of European cities. In 1893 the Norwegian Express route was opened, improving communications with other urban areas along Norway’s northern and western coast. The labour force profited from the 1860s economic expansion, finding jobs in construction and public infrastructure. Trondheim’s connection to the global market made the city economically vulnerable, however. When the ‘Great Depression’ of 1876 hit, it hit Trondheim hard. The city’s exports had made it one of the most rapidly growing urban centres in Norway, but it was soon overtaken by other Scandinavian cities, although it managed to retain its role as the economic heart of its vast hinterland.

Trondheim never became a major centre of industry. In the 1850s, the city boasted just thirty factories, most of which could be characterised as small-scale and artisanal. There were a few larger factories built in the city’s outskirts, but even in 1910 only 15 per cent of the population was occupied in manufacturing industries, whereas a third of the total male labour force was engaged in trade.

Most of the women employed in the labour force worked as domestic servants while others were seamstresses, teachers, nurses or saleswomen. Very few women in Trondheim were recorded as factory workers. The census of 1865 showed that only 22 per cent of women were registered as having an occupation and by the 1910 census, after a substantial decline in the number of adult women recorded as servants, this had fallen even lower to just 12 per cent. There were, however, more women registered as shopkeepers, and even factory owners, in 1910 than had been the case in 1865. Even by the later date, there were few women recorded as workers in the city’s factories.

In Trondheim, general working conditions were not unlike those of Roosendaal. Health and safety legislation was seldom applied to workplaces before the early twentieth century. As a result, many of the poorer workers, and the children in particular, suffered from respiratory diseases such as pneumonia, which was usually a complication of another respiratory infection. Each trade had its own specific risks, but we can safely state that all workplaces—whether factory, construction site or harbour or domestic setting—were hazardous. Entering the labour market, often at a very young age, increased a worker’s risk of suffering from all sorts of accident or catching a variety of diseases. As men were usually assigned the most dangerous tasks, their risk of labour-related injuries, such as burns or fractures, was substantial. While women’s tasks in the workplace were considered to be less dangerous, they nevertheless had to contend with long working hours in far from ideal conditions. They risked injury and illness when working around open stoves, tending the sick, and operating unsafe machinery. Although their work was less well-paid than that of men, the economic value of women’s work in the home, or in Trondheim’s shops and factories was substantial, both for the economy as a whole and for the majority of families.

## Sources

Our comparative analysis is based on two datasets, both containing individual-level CODs from registers of vital events. Although the registers cover considerable periods of time; those in Roosendaal run from 1865 to 1938, those in Trondheim from 1839 to 1911, our analysis here is based on the period 1880–1910. The availability of decennial nominative census data spanning these 30 years allows us to calculate cause-specific and age-specific mortality rates, as the population at risk is known. As explained in section ‘Findings’ below, we excluded those aged less than 10 and more than 70 from our analysis, in order to obtain more robust results.

### Civil registers of Roosendaal

With the introduction of vital registration in 1811, all deaths in the Netherlands were to be recorded in the municipality in which the event took place. From 1865 onwards, individual CODs were to be certified by a trained health professional, usually the local GP, which were then entered in the civil registers by a registrar, as a supplement of the certificate of death. Historical individual COD-data are extremely rare in the Netherlands, being only available for a few towns for short runs of time. The 30-year period 1880–1910 yields a total of 2,354 deaths of individuals aged 10–70. The dataset includes information on the sex, date of birth and date of death of the deceased, however, dividing the data by sex, age and time period produces low numbers of deaths, therefore we must interpret the results with caution.

During our study period, most of those who died in the Netherlands had been treated by a physician, unlike most other parts of Northwest Europe. Local GPs began to treat Dutch families as early as the 1870s.^24^ However, the number of deaths where a doctor had attended the deceased was rather unevenly spread across the country. It was more common to seek medical help in the more affluent coastal, urbanised provinces than it was in the inland provinces. As Roosendaal was situated inland, its data show relatively high numbers of people dying without being seen by a doctor. This had little to do with the fact that there were fewer GPs to attend the sick in the inland regions; the local population were just less inclined to seek medical assistance.^25^ Deaths which occurred without a medical attendant were also spread unevenly within families. The chances of receiving medical treatment were lowest for infants and the elderly and highest for adults. There were more effective therapies available for working-age adults and, as breadwinners, their health was of greater financial importance to their families.^26^

### Parish registers of Trondheim

The Trondheim-database covers the period 1839–1911 and includes data from the burial registers recorded by the priests in each of the city’s churches. Between 1880 and 1910 the registers yield a total of 18,936 burials of persons aged 10–70 with CODs. The dataset provides information on the sex, age, address and occupation of the deceased, although occupation is only registered when the deceased was male or was an unmarried, adult female. When the deceased was a married woman, the occupation of her husband or her father was recorded.

The Trondheim registers indicate that the instructions on how information in them should be recorded changed over time. As early as 1820, the register included a column on the printed form and instructed that deaths involving infectious diseases or accidents should be recorded specifically. From 1877, the instruction stated that all CODs were to be registered and an additional column was added for information whether or not the deceased had been visited by a medical doctor either prior to or after death. The register included church members as well as members from dissenter communities. Early in the nineteenth century legislation had been passed in Norway which meant that priests were required to keep the parish registers not only as record of religious milestones, but also as the basis of counts of vital events which they were to report annually to the authorities who, in turn, used them to compile population statistics. From 1866, the priests’ reports were no longer aggregated counts of events, but were replaced with nominative reports on the individual events, providing more accurate data for statistical purposes. COD information was required for medical statistics; in the early years this was provided by the priest through the nominative reports, but, from 1853 onward, the information was increasingly supplied by medical professionals. From the statistical reports, it would seem that, at least in the nineteenth century, priests were more often present at deaths than doctors were, and were therefore better placed to determine the COD.^27^

The statistical reports clearly show that there was substantial regional variation and a marked rural–urban differential in Norway. Previous studies of infant mortality in rural areas of Norway have revealed that because doctors in rural areas had to travel long distances to reach their patients’ homes, they seldom visited patients. This meant that the certification and registration of CODs was largely in the hands of the priest from the local church.^28^ In the larger towns things were different, as distance was not such an issue, particularly as more doctors lived in the urban areas. As it was relatively easy to find access to medical help in Trondheim, we can assume that the CODs reported in the church registers probably reflect a mix of local priests’ medical knowledge and information provided by a doctor.

The Norwegian Health Law of 1860 directed that every municipality in the country should install a public health committee. In rural areas, the committees’ main priority was to educate the population about standards of personal hygiene, while in urban areas their most critical task was to decrease the number of risks to health. In rural areas the committees tended to consist of public employees, such as teachers and priests, who had been working on health related issues before the 1860-Law was enacted. In the urban areas, however, such committees tended to be more specialised and had greater scientific knowledge so they were more likely to undertake public health and engineering projects. As a result priests were often removed from their position as committee members.^29^ Urban medical professionals took control of COD-registration and were increasingly responsible for the registered CODs. In Bergen, for example, CODs vanished rapidly from the church-registers after 1880. This was not the case in Trondheim. Here, we find the CODs registered by the priest long after 1880. Whether or not he also certified the CODs remains unclear.

## Cause of Death Data

How reliable is historical COD data? Even although modern medical literature still discusses the problems of how to determine the ‘real’ COD, nowadays we assume that medical tests or autopsies can define a COD, usually with great accuracy. A similar level of accuracy cannot be expected when interpreting historical records.^30^ The doctor or priest certifying a COD may not have seen or known the deceased, and would therefore have had to rely on information given by relatives and neighbours, to determine what had caused a death. If an epidemic was in progress, the doctor or priest may have assumed that it had claimed another victim. Medical knowledge was evolving during the late nineteenth century, and this meant that the classification schemes being used with CODs, nosology and medical terminology were constantly changing. Scholars have discussed how changes in a nosology might have affected how diseases were recorded in the registers.^31^ How did the priest or doctor learn of such changes, and did they comply with them? In addition, as the CODs recorded served a statistical purpose, we must examine the extent to which the classification schemes developed to deal with CODs were affected by the epidemiology of different areas. Finally, we are particularly interested in the way in which the CODs recorded varied according to the sex or gender of the deceased. Generally, diseases were surrounded by myths, fears and embarrassment relating to the body and sexuality.

### Coding and classifying causes of death

For coding and classifying all CODs in our datasets, we used a classification scheme entitled Cph1876+.^32^ This classification is hierarchically structured, and is a reworking of a system developed by medical historian Bernabeu.^33^ Unlike the International Classification of Disease (ICD), the standard tool for classifying diseases in current-day societies, our coding and classification tool leaves more room for CODs found in historical sources, and for older views of health. Cph1876+ includes 141 categories, classified into six main groups: infectious diseases; non-infectious diseases; external causes; historic conditions; ill-defined; not reported.

Group 1, infectious diseases, includes five sub-categories. The first of these, infectious diseases transmitted mainly by air and direct human contact, we refer to as the ‘airborne’ diseases. It includes both childhood infections such as smallpox and respiratory infections such as bronchitis and TB. The second sub-category is ‘water- and food-borne diseases’, which include cholera, typhoid fever, and acute digestive diseases such as diarrhoea. The third sub-category covers ‘vector transmitted infectious diseases’. Exanthematic, or louse-borne, typhus and mosquito-borne yellow fever are amongst the diseases in this sub-category, along with puerperal fever, as it is transmitted by human vectors. The fourth sub-category consists of more vaguely described infectious diseases for which the mode of transmission cannot be determined, excluding them from any of the preceding sub-categories. The fifth and final sub-category comprises a group of ‘other specific infections’, including anthrax and tetanus.

Group 2, the ‘non-infectious diseases’, is divided into sixteen sub-categories. These mainly relate to the different parts of the body affected, such as the brain, lungs and the genitourinary system, but also includes categories for deficiency diseases, perinatal pathologies and cancer. Group 3 covers all deaths from all sorts of ‘external causes’, including vehicle accidents, burning and suicide.

Group 4 we have called ‘historic conditions’. The causes assigned to this category, such as ‘malnutrition’, ‘sudden death’, ‘eclampsia’, ‘lack of breastfeeding’, ‘teething’, ‘oedema’, and ‘old age’ have usually been designated as ‘ill-defined’ causes. Recent research has, however, underscored the importance of including these types of condition in historical analyses of CODs. For instance, research has shown that an increase in deaths supposedly caused by a rise in cardiovascular diseases was, in fact, the result of a rebranding of deaths from ‘old age’ to ‘heart disease’, and not a real increase at all.^34^ We are aware that the ‘historic conditions’ category includes a wide variety of diverse CODs which might disturb observed trends over time, but we present them as one group in our analysis. Group 5 contains all CODs which were badly specified, ill-defined, illegible or indecipherable. Group 6 is reserved for those cases where the COD field in the original source was left blank.

By applying the same classification scheme to the CODs registered both in Trondheim and in Roosendaal, this article aims to compare sex- and gender-driven disease patterns and coding practices in the two locales. Although both locations share general West-European norms and values concerning gender, we expect that as well as their local epidemiology, local processes of gender differentiation bound up in each community’s socio-economic structure, labour market and medical practices will influence the CODs registered in each community.

## Findings

Our findings are based on all deaths reported in Roosendaal and all burials registered in Trondheim between 1880 and 1910. We focus on the deaths of those aged 10–70: in Roosendaal we have 2,353 observations (1,235 men; 1,119 women), in Trondheim we have 18,936 observations (9,168 men; 9,625 women).

**Fig. 1:**
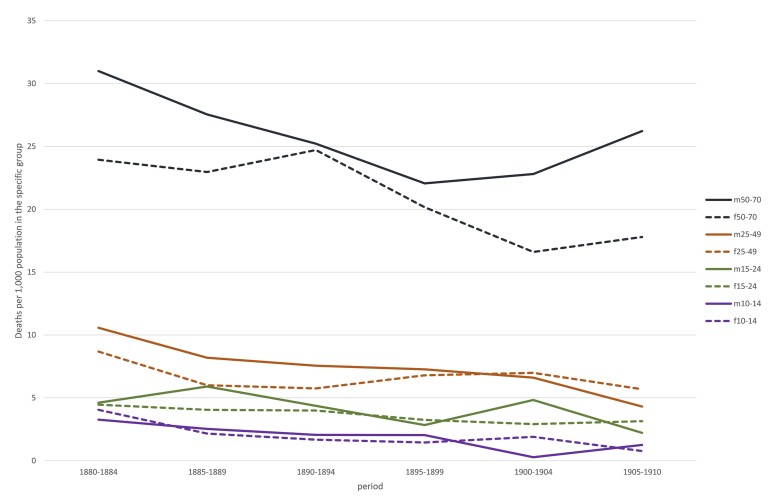
Mortality rates by age and sex, Roosendaal 1880–1910

**Fig. 2:**
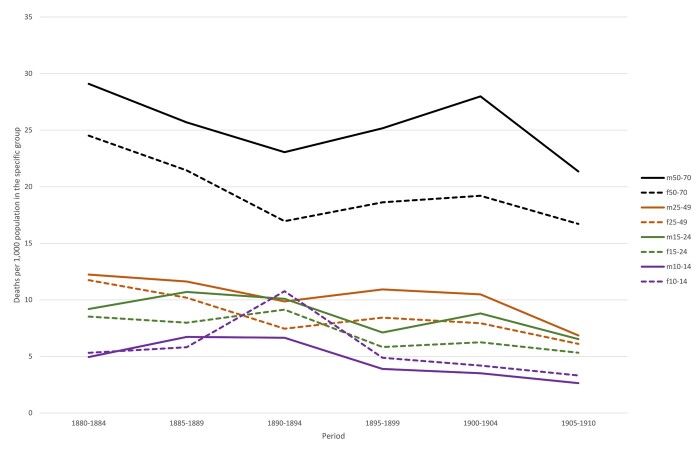
Mortality rates by age and sex, Trondheim 1880–1910

As a first step in our analysis we consider differences in overall mortality between the sexes in each location. In order to calculate sex- and age-specific mortality rates, we used census information to calculate the population at risk.^35^ Detailed census data by age and sex is only available for Roosendaal from 1879 onwards, which meant we could only calculate mortality rates from 1880 onwards. Since the censuses were taken every tenth year, a person aged 0 in the first census had reached the age of 10 in the second census. It is not possible to obtain a reliable estimate of the population at risk amongst those aged less than 10 in census t2. Likewise, we set 70 in census t1 as an upper age threshold for the sake of robustness. Figure 1 and 2, therefore, show location-specific mortality rates for age groups 10–14, 15–24, 25–49 and 50–70, so that the economically active part of the population can be separated from children aged less than 15 and the semi-elderly.

Overall, the figures show signs of a mortality decline in all age groups in both locations. The age and sex specific mortality rates for those aged 10–14 lay well below 5 promille in Roosendaal, which is slightly lower than in Trondheim. For the semi-elderly, the male mortality rates exceeded those of women in both locations: mortality rates for 50–70 year old men fluctuated between 23 and 30 promille, those for women between 17 and 25. The mortality rates in the economically active age groups fluctuated between 4 and 9 promille in Roosendaal and between 6 and 11 in Trondheim.

Figure 1 appears to show excess male mortality in all age groups in Roosendaal but in fact this was only statistically significant (based on confidence intervals of 95 per cent) in the 50–70 age group in the period 1905–1910. Figure 2 suggests that Trondheim had excess female mortality in the 10–14 age group in each quinquennium from 1890–94 onward, but the figures were not statistically significant. All other age groups in Trondheim showed statistically significant excess male mortality from 1890 onwards.

Previous research has shown that sex-specific patterns of disease and mortality become more pronounced in the age groups at which people enter the labour market or start reproducing. The second step of our analysis therefore focused specifically on adolescents and adults aged 15–49. The analysis was based on the deaths of 1,042 individuals (536 men; 506 women) in Roosendaal and 4,711 individuals (2,366 men; 2,343 women) in Trondheim. We expected that the economically active population in the two locations would have better access to doctors and therefore we expected the cause of their deaths to be more accurately reported than those in the other age groups.

**Fig. 3:**
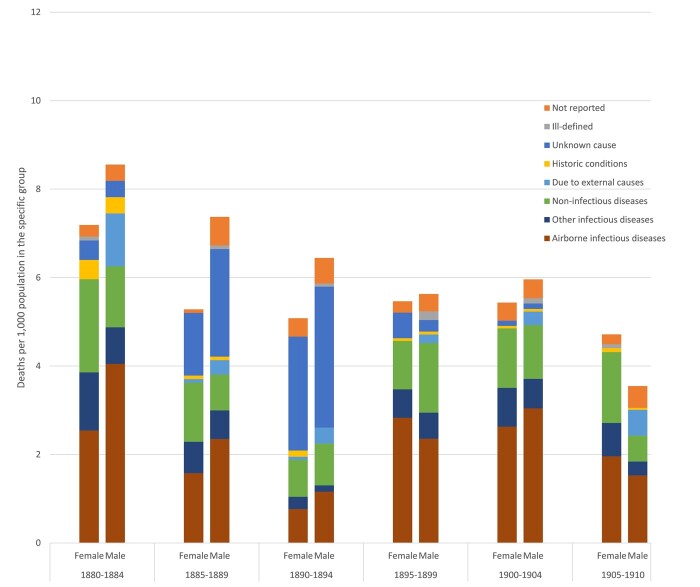
Cause-specific mortality rates for the 15–49 age group, by sex; Roosendaal 1880–1910

**Fig. 4:**
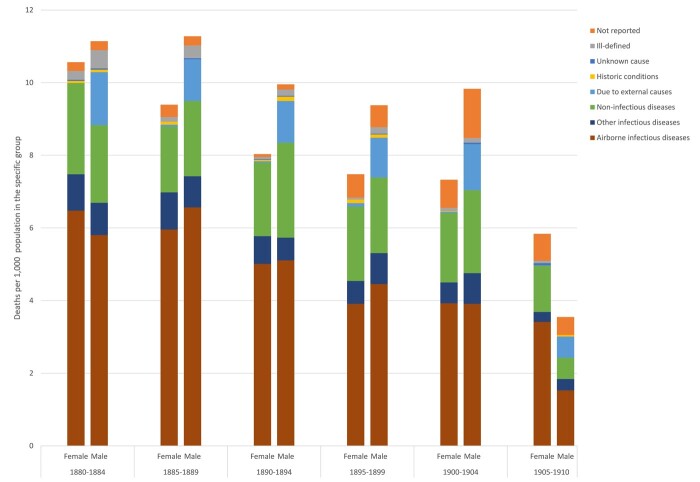
Cause-specific mortality rates for the 15–49 age group, by sex; Trondheim 1880–1910

Figures 3 and 4 show quinquennial mortality rates for the 15–49 age group in Roosendaal and Trondheim, by sex and COD-categories. In both locations, we find that the two infectious diseases categories are, together, significantly more prevalent than all categories of COD. In Roosendaal, we observe that there were differences between men and women in their rates of death from airborne infectious disease, although these were not statistically significant. In the first three five-year periods (1880–94) more men than women died from airborne infectious diseases in Roosendaal, but after 1895 the risk of death from airborne infectious disease was more equal between the two sexes, as women’s risk of dying from these diseases appears to have increased disproportionately. Airborne infectious disease accounted for the largest number of registered deaths in the town, although the pattern is somewhat disturbed by the substantial number of deaths where the cause was recorded as ‘unknown’ in the period between 1885 and 1894, especially amongst men. Although we can only speculate on the exact reason why so many CODs were stated as ‘unknown’, it is highly probable that a considerable proportion of these deaths were actually due to airborne infectious disease. This could certainly explain the relatively low rates of mortality from airborne infectious diseases in 1890–94.

The category of ‘other infectious diseases’, which includes water- and foodborne diseases, was sizeable in the period 1880–84 in Roosendaal, but decreased in relative importance over time. Overall, this category claimed more female than male victims. The non-infectious diseases also showed higher mortality amongst women, at least in 1880–84, 1885–89 and 1905–10. The latter category includes a wide range of CODs, including all the cancers. As expected, men in Roosendaal were more at risk of dying from external causes than women. Interestingly, there were still a few ‘historic conditions’ registered as CODs among men and women aged 15–49 in our study period, although they were more common among women in 1880–84 and 1890–94. There is no distinct pattern in the distribution of ‘ill-defined’ CODs between the sexes. Doctors in Roosendaal left more CODs fields empty or ‘unreported’ for males than for females, but the difference is not statistically significant.

In Trondheim, as is immediately apparent from Figure 4, the airborne infectious diseases were by far the main COD category, among both men and women. As overall mortality rates declined in Trondheim, so did mortality from this type of disease, but the category remained a major COD for both sexes; there was significant difference between male and female rates of mortality from airborne infections. There were no significant sex differentials in mortality from ‘other infectious’ or ‘non-infectious’ diseases. In Trondheim, mortality rates from the non-infectious diseases were higher than those from the other infectious diseases. As expected, men were significantly more at risk of dying as a result of external causes than women. Among men aged 15–49 accidents—predominantly drownings—and suicides were important CODs in the three decades between 1880 and 1910. In both Trondheim and Roosendaal this category was virtually absent among women. External causes may therefore have functioned as a competing COD risk for men; men dying because of an accident would no longer have been at risk of dying from an infectious disease. This scenario would not have applied among women. The broad category of ‘historic conditions’ hardly appears in Trondheim amongst those aged 15–49, but there were a substantial number of cases where the COD was not reported. The number of cases where the priest left the COD field in the burial register blank grew over time. They could have entered the term ‘unknown’, but it is noticeable that this word is hardly ever seen in the Trondheim register.

**Fig. 5:**
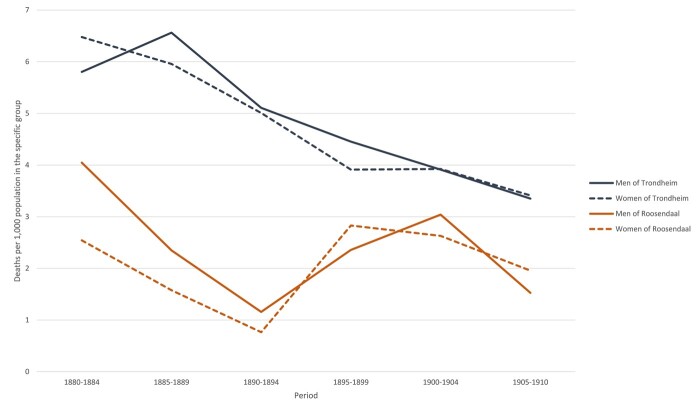
Mortality rates from airborne infectious diseases, for the 15–49 age group, by sex; Roosendaal and Trondheim 1880–1910

As the third step of our analysis we took a closer look at airborne infectious diseases for they, as was shown above, were the major killer in both towns. In our study period, when infectious diseases were still highly prevalent, adolescents and adults were particularly susceptible to the airborne variety. Figure 5 shows mortality rates due to airborne infectious diseases for both sexes in Roosendaal and Trondheim; rates in the latter were higher than in the former. In 1885–89 almost 6.5 men per thousand died from diseases such as TB, bronchitis or pneumonia in Trondheim, whereas the maximum figure in Roosendaal, of almost 4 men per thousand occurred in 1880–84. Comparing the rates of deaths from airborne infectious diseases in 1880–94 and 1905–10, both towns show a declining trend in mortality from airborne infectious diseases, although the mortality rates in Roosendaal increased temporarily in the decade after 1895 before decline set in once more. As discussed above, the steep fall in mortality from airborne infectious diseases in Roosendaal between 1880 and 1894 may have been connected to the sharp rise in the number of CODs which were registered as ‘unknown’ in this period. By 1905–10, male mortality from airborne infections had fallen to 3.5 per thousand in Trondheim and 1.5 per thousand in Roosendaal. The differences in the mortality levels between the two locations are significant for each quinquennium.

Whereas deaths from airborne infectious disease were more pronounced in Trondheim, the rates in both locations show similar sex differentials. In each of the towns men seemed have succumbed to airborne infections more than women, although sometimes the gender gap reverses to give males the advantage. In Roosendaal, male and female trends follow a similar pattern, although before 1895 females were at an advantage, but then at a disadvantage in 1895–99 and 1905–10. In Trondheim, the female mortality advantage, although slight, lasts until the turn of the century. As in Roosendaal, males gain a small advantage in 1905–10. Once confidence intervals are placed on the rates shown in Figures 3–4, however, none of the differences between the sexes in the ‘airborne’ category were statistically significant between 1880 and 1910.

**Fig. 6:**
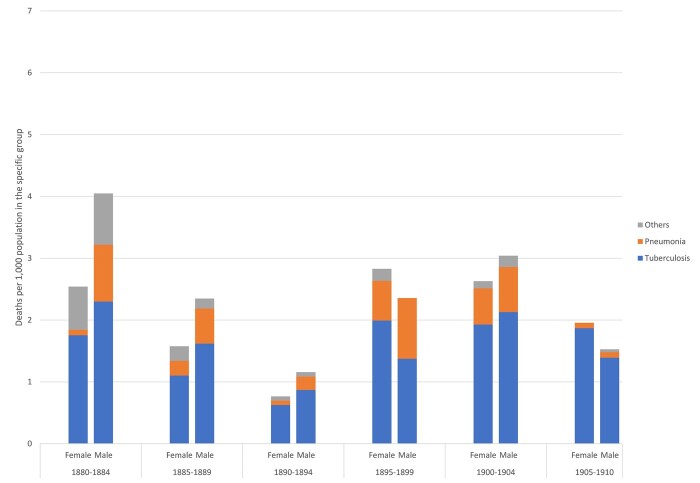
Mortality rates from TB, pneumonia and other airborne infectious diseases, by sex and age, Roosendaal, 1880–1910

**Fig. 7:**
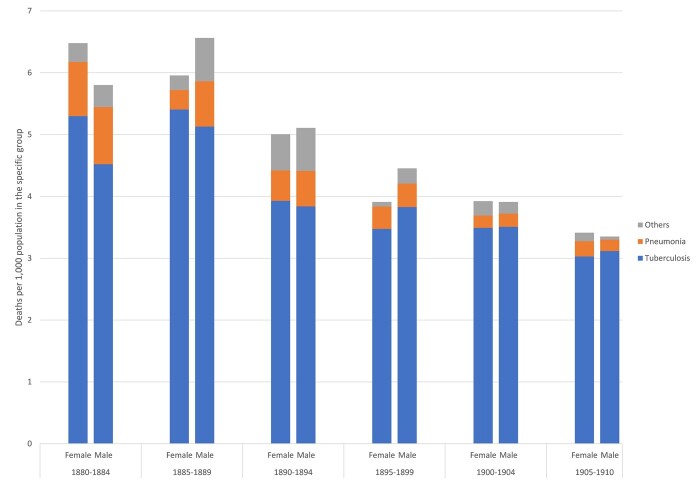
Mortality rates from TB, pneumonia and other airborne infectious diseases, by sex and age, Trondheim, 1880–1910

If airborne infectious diseases were killing so many men and women, especially in Trondheim, which particular diseases in this category were the most lethal? Figures 6–7 shed more light on the specific diseases within the ‘airborne’ category which struck Roosendaal and Trondheim, respectively. Given the number of deaths in the 15–49 age group in each locality, we were able to consider those from TB and pneumonia as separate diseases, but had to combine all other airborne infectious diseases. Of the three, TB was, as expected, the most frequently registered in both locations. Figure 6 shows that in Roosendaal TB exceeded all other airborne diseases to a significant degree. Although the Figure shows male and female differences in mortality from TB and pneumonia in the town, these were not significant as the absolute number of observations was relatively small, once the deaths had been disaggregated into the specific diseases. Taken across the three decades of the study pneumonia appears to have been more lethal for men than women. Generally, TB also appears to have claimed more male victims than female.

In Trondheim, rates of mortality from airborne infectious disease were driven to a striking degree by deaths from TB. Figure 7 shows that, during the three decades, TB was the major killer within the ‘airborne’ category of both men and women. This is particularly the case after 1900, when deaths from airborne diseases occur almost exclusively from TB. Pneumonia was also an important killer, but was always overshadowed by TB. Pneumonia did not show a distinct sex/gender pattern as was the case in Roosendaal: however, the population of Trondheim experienced, at least in the first decade of the twentieth century, a period in which pneumonia primarily claimed female victims. In the period 1885–94 pneumonia showed higher male mortality rates while female TB mortality rates exceeded male over the same period. In 1895–99 male TB mortality rates were higher, but around the turn of the century, male and female TB mortality returned to similar rates. In sum, as in Roosendaal, we find that TB was the dominant disease in the category of airborne infections among both men and women in Trondheim. Overall, we do not observe significant sex/gender differences in airborne infectious disease mortality in both locations.

The fourth and final step of our analysis focused on local COD registration practices. We tried to assess whether the registration process and the nature of the CODs recorded were equally specific, elaborate or varied for both sexes. For this analysis we considered all the deaths occurring to men and women aged 15–49 in both locations.

In Roosendaal there were 1,042 relevant deaths (506 female; 536 male). The women that died in Roosendaal were assigned a total of 195 COD terms, and the men were given 181. The same type of disease may be referred to by different terms but would be assigned to the same ‘COD’. For example, ‘*longtering*’ and ‘tuberculosis pulmonum’, both refer to respiratory TB. The absolute numbers in Trondheim were of a different order of magnitude; the deaths of 2,343 women were assigned to 639 distinct COD terms, while the 2,366 male deaths included 809 COD terms. Proportionately, the women of Roosendaal had a greater variety of CODs recorded than the women of Trondheim. Men in the two locations had a similar variety of causes recorded. We should note that multiple CODs were not routinely recorded in Roosendaal; we found only four such cases. In Trondheim, however, the registration of more than one COD was quite common, although the deaths of men were more likely to be registered with multiple CODs: 6.5 per cent of all female deaths were recorded with 2, 3 or 4 causes listed as contributing to their death. There were 7.4 per cent of all male deaths similarly registered. Our analysis below is based on the first COD given for any one individual.

Table 1 shows the top 30 recorded COD terms for each sex in Roosendaal and Trondheim. The table shows the frequency of each cause and the cumulative percentage contribution of the 30 causes to the total deaths for each sex. In Roosendaal, the top 30 registered CODs account for 60.5 per cent of all female deaths reported. We have to bear in mind that ‘unknown’ (‘*onbekend*’) deaths are one of the top 30 causes, in fact *the* top cause, in the Roosendaal lists, and ‘not reported’ is the second most common COD for both sexes in Trondheim. The top 30 CODs account for 67.7 per cent of the reported male deaths in Roosendaal. In other words, there was greater variety of CODs reported amongst women. In Trondheim, the difference between the sexes shows the reverse: the top 30 registered CODs account for 64.3 per cent of the female and 55.9 of the male deaths. Although Table 1 only shows the top 30, there were a substantial number of deaths where only one person was assigned a particular COD. In Trondheim this was the case for 513 women (22 per cent) and 672 (28 per cent) men; while in Roosendaal 138 women (27 per cent) and 129 men (24 per cent) were given a COD that only appeared once.

**Table 1.** Cumulative frequency of causes of death registration, by sex, for age group 15–49, Roosendaal and Trondheim, 1880–1910

Female (Roosendaal)			Male (Roosendaal)			Female (Trondheim)			Male (Trondheim)		
Causes of death (standardised)	count	Cum- perc	Causes of death (standardised)	count	Cum- perc	Causes of death (standardised)	count	Cum- perc	Causes of death (standardised)	count	Cum- perc
Onbekend	68	13.4	Onbekend	84	15.7	Lungetæring^*^	289	12.3	Lungetæring^*^	247	10.4
Tuberculosis pulmonum^*^	41	21.5	Niet vermeld	44	23.9	Not reported	151	18.8	Not reported	138	16.3
Phthisis pulmonum^*^	34	28.3	Phthisis pulmonum^*^	41	31.5	Phthisis pulmo^*^	130	24.3	Phthisis pulmo	99	20.5
Niet vermeld	26	33.4	Tuberculosis pulmonum^*^	35	38.1	Tuberculosis pulmo^*^	127	29.7	Tuberculosis pulmo^*^	99	24.6
Phthisis pulmonalis^*^	13	36.0	Pneumonie	19	41.6	Tuberculosis pulmonalis^*^	115	34.7	Tæring^*^	97	28.7
Tuberculose van de longen^*^	13	38.5	Tuberculosis^*^	13	44.0	Tæring^*^	103	39.1	Tuberculosis pulmonalis^*^	76	32.0
Peritonitis	9	40.3	Longtering^*^	12	46.3	Phthisis pulmonalis^*^	79	42.4	Lungebetennelse	70	34.9
Febris puerperalis	8	41.9	Tuberculose van de longen^*^	12	48.5	Lungebetennelse	47	44.4	Lungetuberkulose^*^	45	36.8
Tuberculosis^*^	8	43.5	Carcinoma ventriculi	11	50.6	Lungetuberkulose^*^	45	46.4	Druknet	40	38.5
Carcinoma mammae	6	44.7	Verdrinking	10	52.4	Brysttæring^*^	34	47.8	Phthisis pulmonalis^*^	35	40.0
Pokken	6	45.8	Pneumonia crouposa	7	53.7	Pneumonia	32	49.2	Difteri	30	41.3
Vitium cordis	6	47.0	Gastroenteritis	6	54.9	Tuberculosis^*^	32	50.5	Pneumonia	27	42.4
Morbus brighti	5	48.0	Phthisis pulmonalis^*^	6	56.0	Nervefeber	30	51.8	Nyresykdom	26	43.5
Pneumonia chronica	5	49.0	Pneumonia chronica	6	57.1	Hjertefeil	28	53.0	Tuberculosis^*^	26	44.6
Pneumonie	5	50.0	Pneumonie crouposa	6	58.2	Phthisis^*^	23	54.0	Magekreft	23	45.6
Carcinoma	4	50.8	Meningitis	5	59.1	Barselfeber	22	54.9	Nervefeber	23	46.5
Icterus gravis	4	51.6	Pneumonie chronica	5	60.1	Difteri	22	55.9	Phthisis^*^	23	47.5
Longtering^*^	4	52.4	Aneurisma	4	60.8	Febris puerperalis	20	56.7	Hjertefeil	22	48.4
Peribronchitis chronica	4	53.2	Apoplexia	4	61.6	Lungetuberculose^*^	20	57.6	Brysttæring^*^	20	49.3
Peritonitis acuta	4	54.0	Insufficintia valvulae mitralis	4	62.3	Magekreft	20	58.4	Hjernebetennelse	19	50.1
Phthisis^*^	4	54.7	Peritonitis	4	63.1	Magebetennelse	18	59.2	Morbus brights	17	51.1
Septichaemie	4	55.5	Phthisis^*^	4	63.8	Morbus brights	17	59.9	Tyfus	17	51.8
Ziekte van bright	4	56.3	Verwonding	4	64.6	Cancer ventriculus	15	60.6	Lungetuberculose^*^	15	52.4
Apoplexia	3	56.9	Bronchitis	3	65.1	Hjernebetennelse	15	61.2	Tuberkulose^*^	14	53.0
Carcinoma hepatis	3	57.5	Hydrothorax	3	65.7	Hjertelammelse	14	61.8	Apoplexia cerebri	12	53.5
Carcinoma uteri	3	58.1	Pokken	3	66.2	Kreft	13	62.4	Meningitis	12	54.1
Carcinoma ventriculi	3	58.7	Algemeene tuberculose^*^	2	66.6	Nyresykdom	13	62.9	Vitia organ cordis	12	54.6
Causa ignota	3	59.3	Bloedspuwing(^*^)	2	67.0	Tuberkulose^*^	13	63.5	Cancer ventriculus	11	55.0
Endocarditis chronica	3	59.9	Brightsche ziekte	2	67.4	Cancer uteri	11	63.9	Hjerneslag	11	55.5
Gastroenteritis	3	60.5	Bronchitis acute	2	67.7	Hjerneslag	9	64.3	Hjertelammelse	10	55.9

* refers to TB

If we focus attention on the top 30 registered CODs for women in Roosendaal, after the large group of ‘unknown’ causes the top 10 female CODs are dominated, as expected, by a variety of terms associated with TB, particularly respiratory TB. There are at least seven COD terms referring to this form of TB in the top 30, four of them in the top 10. When registering such deaths the registrar most often gave the Latin or Greek terms for respiratory TB: tuberculosis pulmonum or phthisis pulmonum but ‘*tuberculose van de longen*’ was also used. Typically female diseases such as puerperal fever and breast cancer also appear in the top 10 and a relatively high number of different types of cancer or tumours were included in the top 30. Cases where the CODs were stated to be ‘unknown’ are also at the top of the list for men in Roosendaal. This is unexpected, as the men in the 15–49 age group would have been the backbone of the local economy, and as such ought to have had greater access to medical care and diagnoses. They too had a variety of terms for respiratory TB recorded; at least five alternatives appear on the male list, six if we add the more symptomatic description of TB, *bloedspuwing* (‘coughing up of blood’). Remarkably, ‘drowning’ appears tenth on the list, highlighting the important role accidents played in male mortality; injuries (*verwondingen*) also appear further down the male top 30, which also features several terms for pneumonia and bronchitis, both in the airborne infectious disease category.

Like Roosendaal, a high number of deaths in Trondheim had an ‘unknown’ COD, but unlike such cases in Roosendaal, this was because the COD field in the registration had been left blank. TB dominated the top 10 for both sexes in the Norwegian town. Respiratory TB was again the most common form registered, with a total of eight distinct terms being used, of which the Norwegian word ‘*lungetæring*’ was the most frequent. For both sexes, twelve out of the top 30 CODs related to TB. Other female CODs listed include pneumonia (*lungebetennelse*), typhoid fever (*nervefeber*), heart failure (*hjertefeil*), diphtheria (*difteria*), puerperal fever (*barselfeber*), and cancer. Forty men are recorded as dying from the one term ‘drowning’, putting it in ninth place. As will be explained below, these forty observations were just a small proportion of the 195 total instances of drowning in the dataset.

The classification of CODs used in the official Dutch medical statistical reports from 1875 onwards meant that only 34 categories of COD were reported. Some of these were specific diseases which, we suspect, were included because of their high frequency and importance to public health. Infectious diseases such as measles were each assigned their own category, for example, while other categories referred to much broader groups of diseases such as ‘diseases of the respiratory organs’. A closer look at the CODs recorded in the registers reveals the enormous variety of terms, both in Latin and in the vernacular, that contributed to the aggregated categories.

The picture in Norway was quite different. Even by 1861, the series of health statistics, published from 1853 onwards, had revealed some weaknesses and problems in the process of COD registration, categorisation, and publication of statistics. Medical men were invited to make full use of a pre-printed form, first issued in 1852, when making their medical annual reports to the Ministry of the Interior. However, the Ministry soon declared this reporting to be incomplete, so that properly detailed and comparable statistics could not be published. The doctors, it appeared, often failed to specify the sex or age of the deceased and had not been provided with a standardised nomenclature for diseases. Consequently, the Ministry issued a ‘*circulære*’ in 1861 detailing several measures which, it was thought, would improve the COD statistics. The circular letter defined a total of 122 CODs and provided instructions on which Norwegian and Latin terms should be used to describe them.^36^ Many of the 122 CODs contained subdivisions. For example, the specific organ affected had to be noted in cases of ‘tuberculosis’ and ‘cancer’. In Trondheim, where priests were registering deaths, to what extent did they follow the 1861 COD nomenclature? Around 60-70 per cent of the terms used to report male and female CODs in the Trondheim registers in 1880-1910 can be traced back to the 1861 nomenclature. Some priests preferred to register the COD in the vernacular, others in Latin, and some applied a mix of languages. There were two striking ways in which the priests in Trondheim failed to follow the rules on how CODs should be assigned to the categories in the nomenclature. First of all the priests tended to report the underlying symptoms of a COD, and secondly they gave many more details of events at or leading up to a death, which must have been provided either by persons close to the deceased or by a medical doctor. Doctors were not expected to provide such detail; they were instructed to use the terms set out in the nomenclature, so the fuller descriptions given by the priests offer an interesting glimpse into contemporary registration practices. The details given in the registers mean that there are many single-frequent CODs in our datasets, and we find some interesting differences in those provided for men and women in Trondheim. First of all a large proportion of the single-frequent causes were detailed descriptions of accidents; 271 observations, most relating to male deaths, made up 40 per cent of the single-frequent COD terms. Accounts of drownings accounted for 155 of these observations, i.e. 79 per cent of all male drownings. When someone drowned the priest typically registered where and how it happened, as the following example illustrates; ‘Drowned, accident. The corpse was found in the harbour. It is presumed [he] was sleeping on a bench on the pier, and rolled off and into the water’ (own translation). When we look at typically female CODs, such as those related to childbirth, or breast and uterine cancers, there were 141 observations but only 41 had a frequency of one (29 per cent), compared to 79 per cent single-frequent male drownings. A less clustered pattern of specific groups of CODs among women is also interesting. It indicates a greater variance between the sexes in how ‘narrative’ CODs are recorded within the already detailed registration.

**Fig. 8:**
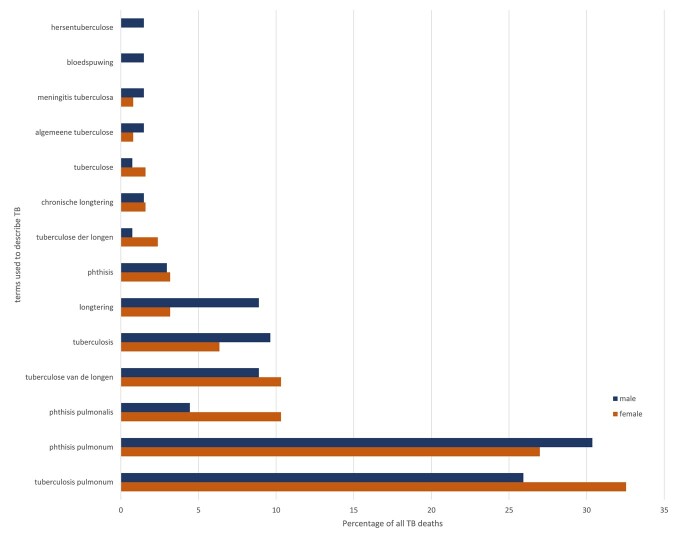
Individual terms used to describe cause of death from TB, percentage by sex, Roosendaal, 1880–1910

**Fig. 9:**
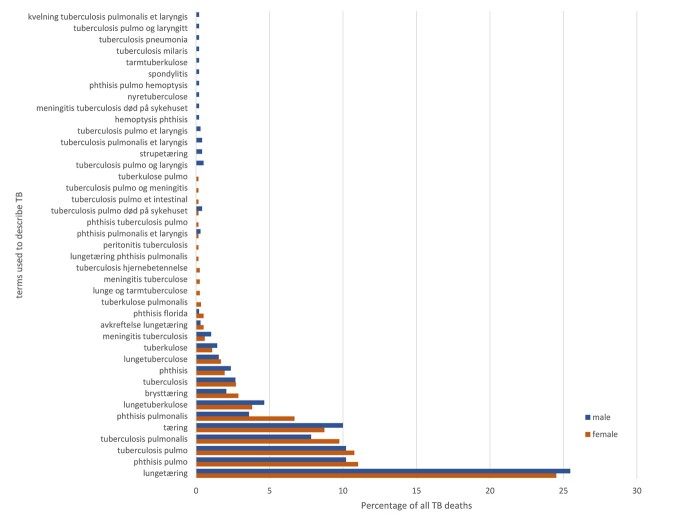
Individual terms used to describe cause of death from TB, percentage by sex, Trondheim, 1880–1910

Finally, we turn again to TB mortality, this time to look at the individual CODs recorded. The variety of terms used to describe diseases which fall into the ‘tuberculosis’ COD category are shown in Figures 8–9. The figures list the percentage of terms used to describe various forms of TB with a frequency higher than one of the total numbers of TB deaths by sex. Overall, among the deaths in Roosendaal there were 35 distinct terms for women and 23 terms for men which referred to some type of TB. As shown in Figure 8, respiratory TB was the most often reported term for both sexes. In total seven terms were used for this form of TB, including the Latin terms ‘tuberculosis pulmonum’ and ‘*longtering*’ in the Dutch vernacular. The vernacular term was used four out of seven times. 10 per cent of the male TB deaths had no description of the disease beyond the one word, ‘tuberculosis’. This only counted for 6 per cent among the women. Of the twelve terms regarding women, six were in the vernacular. Eight of the terms used for TB deaths amongst men were given in the vernacular. Among the one-frequency terms, not shown in Figure 8, many women dying from TB in Roosendaal were reported to have TB in organs or parts of the body other than their lungs, such as the kidneys. There were very few cases of abdominal TB. Among men, TB of the larynx, the brain and the cerebral membrane all appear.

Figure 9 shows the percentage of terms used to describe CODs from TB with a frequency higher than one of the total numbers of TB deaths, for the Trondheim women and men respectively. This constitutes about 90 per cent of the total registered terms with a TB COD. Notably, about 10 per cent of the TB terms for both sexes were single-frequent CODs. Overall, there were 148 cases, out of the total 1,179 female TB deaths, where a single-frequent term was used to describe the COD, and 147 cases out of 970 male TB deaths. For both sexes, the most frequently reported terms were *lungetæring*, phthisis pulmo(nalis), tuberculosis pulmo(nalis), and *tæring*, which all refer to respiratory TB. There was also a high number of terms referring to TB in other parts of the body for both sexes. This may have resulted from the way that the official nomenclature required specification which part of the body was infected in case of TB death. There were 121 terms (10 per cent) describing deaths from TB amongst women which were only encountered once, and 116 such terms (12 per cent) amongst men (not in Figure 9). Again, as in Roosendaal, it seems that priests in Trondheim tended to register additional information. For example, one such entry reads: ‘Respiratory TB. She arrived one year ago from Chicago, where she got married, in order to regain her strength’ (own translation). When it came to the use of vernacular versus Latin or Greek terms, deaths in Trondheim were as likely to be recorded in Norwegian, using *lungetæring*, *tæring*, *brysttæring* and *lungetuberkulose*, as they were in Latin. Sometimes a mix of languages was used. In Trondheim, just as in Roosendaal, it would seem, vernacular terms for TB and its many variations, were deeply embedded in medical descriptions of CODs.

## Discussion

The aim of this article has been to investigate how a person’s sex, or the ideas concerning gender roles and the idea of ‘gendered disease’ were reflected in late nineteenth- and early twentieth-century mortality patterns. We based our analysis on the individual-level COD records from two Northwest-European urban communities; Trondheim in Norway and Roosendaal in the Netherlands. Our analysis followed a novel four-step path, in which we moved from an analysis of cause-specific mortality rates to an analysis of the details included in the individual-level CODs, enabling us to take full advantage of the richness of the data. In practice, when using the historical data, it proved extremely difficult to separate the effects of ‘sex’ and ‘gender’, particularly as biology and behaviour often interact. What we observe in the data is a combination of sex-specific mortality patterns, and the way ideas of sex and gender influenced how medical diagnoses were recorded.

Overall, the results show signs of mortality decline in all age groups in both locations. The economically active population, which formed the main focus of this article, saw their mortality rates fluctuate between 4 and 11 per thousand in both locations, although the rates were slightly higher in Trondheim. When we analysed mortality by sex and age our findings confirmed those of most previous studies^37^; excess male mortality was the norm in both communities, although the difference between males and females in each age group in Roosendaal was not statistically significant. In Trondheim, each of the over-14 age groups showed statistically significant excess male mortality in the 1890s and 1900s. While our findings show a female survival advantage, the mechanisms behind this trend in each of the locations is of particular interest. Like boys, girls over the age of 14 entering the labour market encountered harsh working conditions in both towns. Labour market participation was forging new gender roles for men and women during their reproductive years. This affected male and female cause-specific mortality rates in different ways, but female mortality remained lower than that of men. Young women leaving the labour market to care for their families and take on domestic duties still faced risks to their health from poor living conditions and crowded housing. Nevertheless, their mortality was lower than that of working-age men. Women in both locations were most likely to die from airborne infectious diseases, respiratory TB in particular. Hinde suggested that TB was the result of women’s work in overcrowded factories,^38^ but this cannot explain the high rates of this disease in Trondheim, where women were seldom employed as factory workers. Houses as well as workplaces were cramped, dusty and damp providing excellent conditions in which airborne disease organisms could be transmitted. We assume that it was a combination of such housing conditions and the expectation that women would undertake the care of sick family members that resulted in the high numbers of TB related deaths in the town. This interpretation coincides with McFarlane’s findings in Scotland^39^; remaining at home, especially after marriage, did not prevent women in our two locations from catching and succumbing to airborne infectious diseases. Based on gendered societal norms, we had expected to find evidence to support the ‘bargaining-nutrition’ theory in Trondheim: however, this did not prove to be the case.

Airborne infectious disease was also the main registered COD category among men in both towns: there were no significant differences between the male and female rates of mortality from these diseases in either location, although the rates were significantly higher in Trondheim throughout our period. The higher rates of mortality from airborne infectious disease in Trondheim were driven by high rates of TB. As well as working conditions in factories which predisposed men to respiratory diseases, the labour markets in both localities also presented other types of hazard. In both towns men were more likely to die from external causes such as accidents and work-related injuries. This confirms previous findings and adds to the literature, as previous studies have mostly been based on twentieth-century statistics.^40^ Men in nineteenth-century urban centres had to work with new machinery and new forms of transportation, and the associated hazards are reflected in the high proportion of male casualties. As Trondheim was surrounded by sea and fjords, accidental deaths involved a high proportion of drownings. We suspect that deaths from external causes may have functioned as a competing risk for men in mortality terms: men who died as the result of an accident were no longer at risk of dying from an infectious disease.

We found that TB was the most frequently registered COD. As the number of TB deaths reflected only a small proportion of all those afflicted by this disease, which was characterised by its infectious nature and chronic progression, it must have been a significant burden on the daily lives of the populations of Trondheim and Roosendaal. TB mortality was higher in Trondheim, particularly among those aged 15–49. Various types of TB dominated the COD frequency tables in both locations. Diseases which typically affect females lay alongside TB at the top of the COD frequency tables for women. In both locations, puerperal fever and breast cancer were amongst the top 10 most frequently recorded CODs. These two diseases would have presented themselves in a clear and unambiguous manner to the doctors and to the priests registering the deaths, ensuring that they were registered as the COD.

Finally, we investigated the registration practices in our two locations to assess whether COD reporting was equally detailed for both sexes. In Roosendaal, there was greater variation in the records of women’s deaths, but in Trondheim the CODs given for men were more varied. Using respiratory TB as an example, we showed that a substantially greater variety of descriptions of this disease were used in Trondheim compared to those in Roosendaal. This could be a result of the relatively lower number of observations in Roosendaal, however, we find the high proportion of detail in the COD narratives in Trondheim striking, despite the availability of a nomenclature. Furthermore, the recording of multiple CODs was quite frequent in Trondheim, but more or less absent in Roosendaal. Here, a trained medical practitioner was responsible for the diagnosis of COD reported in the registers, whereas in Trondheim the local priest did most of the recording. We suggest, therefore, that the differences both within and between the two locations in the CODs recorded are a consequence of the professional backgrounds of the individuals responsible for recording the CODs. We further suggest that the priests in Trondheim were not under the same restraints as medical practitioners and could therefore act autonomously. They certainly appear to have thought it valuable to add as much information as possible about the deceased and how they died when compiling the records.

## Conclusion

The overall findings show differences in male and female COD-patterns which could be the result of either variations in biological risk, the gendering of disease, or local registration practices. Our main conclusion is that local registration practices contributed highly to the observed differences and that our study does not find a marked gendering of disease. Our findings show excess male mortality among age group 15–70. Airborne infectious diseases were responsible for most deaths in both cities, but did not show a distinct gender pattern. Also, TB appeared to be more location-specific than gender-specific. However, the level of variation and specification in TB COD terms was higher among women in both locations.

## Footnotes

1 Alice Reid *et al.*, ‘‘A Confession of Ignorance’: Deaths from Old Age and Deciphering Cause-of-death Statistics in Scotland, 1855-1949’, *The History of the Family*, 2015, 20, 320–44.

2 Barbara Blatt Kalben, *Why Men Die Younger: Causes of Mortality Differences by Sex* (Schaumburg: Society of Actuaries, 2002), 39–40; M. Dinges and A. Weigl, eds, ‘Gender-Specific Life Expectancy in Europe 1850-2010’, special issue *Medizin, Gesellschaft und Geschichte—Beihefte*, 2016, 58; Virginia Zarulli *et al.*, ‘Women Live Longer than Men even During Severe Famines and Epidemics’, *PNAS*, 2018, 115, E832–840.

3 M. Luy, ‘Causes of Male Excess Mortality: Insights from Cloistered Populations’, *Population and Development Review*, 2003, 29, 647–676.

4 Ingrid Waldron, ‘Factors Determining the Sex Ratio at Birth’, in United Nations, *Too young to die: genes or gender* (New York: United Nations, 1998) 53–63, 61; Ingrid Waldron, ‘Sex Differences in Infant and Early Childhood Mortality, Major Causes of Death and Possible Biological Causes’, in United Nations, *Too**Young to**Die:**Genes or**Gender* (New York: United Nations, 1998), 64–83, 78.

5 Constance Holden, ‘Why Do Women Live Longer Than Men?’, *Science*, 1987, 238, 158–60; Waldron, ‘Sex differences’, 65; James B. Hamilton and Gordon E. Mestler, ‘Mortality and Survival: Comparison of Eunuchs with Intact Men and Women in a Mentally Retarded Population’, *Journal of Gerontology*, 1969, 24, 395–411; K. L. Margolis *et al.*, ‘Effect of Oestrogen Plus Progestin on the Incidence of Diabetes in Postmenopausal Women: Results from the Women’s Health Initiative Hormone Trial’, *Diabetologia*, 2004, 47, 1175–1187; G.L. Drevenstedt *et al.*, ‘The Rise and Fall of Excess Male Infant Mortality’, *PNAS*, 2008, 105, 5016–5021; Zarulli *et al.*, ‘Women Live Longer’.

6 Linda L. Lindsey, *Gender Roles: A Sociological Perspective* (London and New York: Routledge, 2016, 6th edn) 4–5.

7 Richard G. Rogers *et al.*, ‘Social, Behavioral, and Biological Factors and Sex Differences in Mortality’, *Demography*, 2010, 47, 555–78.

8 `Fanny Janssen and Frans Van Poppel, ‘The Adoption of Smoking and Its Effect on the Mortality Gender Gap in the Netherlands: A Historical Perspective’, *Biomed Research International*, 2015, ID 370274.

9 Angélique Janssens, *Sekse, gender en de dood* (Maastricht: Inaugural speech, 2016), 20.

10 Amartya Sen, ‘More Than 100 Million Women Are Missing’, *The New York Review of Books*, December 1990; F. van Poppel, J. Schellekens and E. Walhout, ‘Oversterfte van jonge meisjes in Nederland in de negentiende en eerste helft twintigste eeuw’, *Tijdschrift voor Sociale en Economische Geschiedenis*, 2009, 6, 37–69, 38–42; R. E. Kennedy, *The Irish:**Emigration*, *Marriage, and**Fertility* (Berkely: University of California Press, 1973), 41–65; D. Tabutin, ‘La submortalité feminine en Europe avant 1940’, *Population (INED)*, 1978, 33, 121–148; I. Devos, ‘Te jong om te sterven. De levenskansen van meisjes in België omstreeks 1900’, *Tijdschrift voor Sociale Geschiedenis*, 2000, 26, 55–75; Kirsty McNay, Jane Humphries and Stephan Klasen, ‘Excess Female Mortality in Nineteenth-Century England and Wales: A Regional Analysis’, *Social Science History*, 2005, 29, 649–681; A. Janssens, M. Messelink and A. Need, ‘Faulty Genes or Faulty Parents? Gender, Family and Survival in Early and Late Childhood in the Netherlands, 1860-1900’, *The History of the Family*, 2010, 15, 91–108.

11 Dominique Tabitin and Michel Willems, ‘Differential Mortality by Sex from Birth to Adolescence: The Historical Experience of the West (1750-1930), in United Nations, *Too young to die: genes or gender* (New York: United Nations, 1998), 17–52, 42 and 44.

12 Angélique Janssens and Elien van Dongen, ‘A Natural Female Disadvantage? Maternal Mortality and the Role of Nutrition Related Causes of Death in the Netherlands, 1875-1899’, *Low Countries Journal of Social and Economic History*, 2018, 14, 84–115.

13 N.M. McFarlane, *Tuberculosis in Scotland, 1870-1960* (Glasgow: University of Glasgow, 1990), 209.

14 A. Hinde, ‘Sex Differentials in Phthisis Mortality in England and Wales, 1861-1870’, *The History of the Family*, 2015, 20, 1–27; A. Reid and E. Garrett, ‘Mortality, Work and Migration. A Consideration of Age-specific Mortality from Tuberculosis in Scotland, 1861-1901, *Historical Life Course Studies*, 2018, 6, 111–132.

15 Ornella Moscucci, *Gender and Cancer in England, 1860-1948* (London: Palgrave McMillan, 2016), 1; Tammy Duerden Comeau, *The**Gendered**Sub-text of**Medical**Discourse on**Cancer in the**Nineteenth**Century* (unpublished PhD thesis, The University of Western Ontario, 2004); G. Blok, E. Buchheim and E.F. Jonker, *Gender en Gekte. Jaarboek voor Vrouwengeschiedenis* (Amsterdam: Amsterdam University Press, 2010).

16 Duerden Comeau, *The gendered sub-text*, 3–12; Roberta McGrath*, Seeing Her Sex: Medical Archives and the Female Body* (Manchester: Manchester University Press, 2002); Christopher E. Forth, *Masculinity in the Modern West: Gender, Civilization and the Body* (Basingstoke: Palgrave MacMillan, 2008); W.D. Churchill, ‘The Medical Practice of the Sexed Body: Women, Men and Disease in Britain, Circa 1600-1740’, *Social History of Medicine*, 2005, 1, 3–22.

17 L.J P. Van Gastel, *Roosendaal tussen platteland en stad, 1770-1900* (Tilburg: Stichting Zuidelijk Historisch Contact, 1995), 52.

18
*Ibid*., 189.

19 J.J.A.M. Gorisse, *Tachtig jaar Kruisvereniging Roosendaal: 1916-1996* (Roosendaal: Signifikant, 1996), 10–11.

20
*Ibid*., 11.

21 Van Gastel, *Roosendaal*, 144.

22 R. Danielsen, ‘En Exempelløs Fremgang: 1880-1920’, in Grethe Authen Blom *et al.*, eds, *Trondheims historie 997-1997* (Trondheim: Universitetsforlaget, 1997).

23 Jørn Sandnes *et al.*, *Trondheim, One Thousand Years in the City of St. Olav* (Trondheim: Tapir akademisk forlag, 2002), 37–50.

24 W. Rutten, ‘Mortaliteit en medicalisering. Een regionaaldifferentiële analyse van de sterfte zonder geneeskundige behandeling in Nederland (ca 1870–1900)’, *Holland*, 1985, 17, 138.

25
*Ibid*.

26 Willibrord Rutten, ‘Ongelijke behandeling binnen het gezin. Een onderzoek naar de leeftijdsverschillen in de kans op geneeskundige hulp in Nederland (ca. 1870-1900)’, *A.A.G. Bijdragen*, 1986, 245–266.

27 A.G. Pedersen, ‘Dødelighet og dødsårsaker’, in R.R. Bore, ed, *På liv og død. Helsestatistikk i 150 år* (Oslo: Statistisk sentralbyrå, 2007), 49.

28 E. Fure, ‘Dødsårsaker i Asker og Bærum på 1800-tallet’, in E. Fure, *En besyndelig Regelmessighet. Dødeligheten i Asker og Bærum på 1800-tallet med særlig vekt på spedbarnsdødeligheten* (doctoral dissertation, University of Oslo, 2004), 3.

29 Aina Schiøtz and Maren Skaset, Folkets helse—landets styrke 1850-2003. Det offentlige helsevesen i Norge 1603-2003, 2 (Oslo: Universitetsforlaget, 2003), 45–46.

30 George C. Alter and Ann G. Carmichael, ‘Reflections on the Classification of Causes of Death’, *Continuity and Change. A Journal of Social Structure, Law and Demography in Past Societies*, 1997, 12, 169–173; Günther B. Risse, ‘Cause of Death as a Historical Problem’, *Continuity and Change. A Journal of Social Structure, Law and Demography in Past Societies*, 1997, 12, 175–188; K. Codell Carter, ‘Causes of Disease and Causes of Death’, *Continuity and Change. A Journal of Social Structure, Law and Demography in Past Societies*, 1997, 12, 189–198; Frans Van Poppel and Jitse P. Van Dijk, ‘The Development of Cause-of-death Registration in the Netherlands, 1865-1955’, *Continuity and Change. A Journal of Social Structure, Law and Demography in Past Societies*, 1997, 12, 265–87; France Mesle, ‘Classifying Causes of Death According to an Aetiological Axis, *Population Studies*, 1999, 53, 97–105; Alice Reid and Nynke van den Boomen, ‘The Faces of Death: Regional Differentiation in Cause-specific Mortality in the Past’, *The History of the Family*, 2015, 20, 309–19.

31 Alice Reid *et al.*, ‘A confession’.

32 B. Revuelta-Eugercious and A. Løkke, ‘Infections and Poverty’ (ESSHC, Valencia, 2016).

33 Josep Pep Bernabeu, Diego Ramiro Farñas, Alberto Sanz Gimeno and Elena Robles González, ‘El Análisis Histórico de La Mortalidad Por Causas. Problemas y Soluciones’, *Revisita de Demografia Histórica*, 2003, 21,167–93.

34 Reid *et al.*, ‘A confession’, 330.

35 Census information provided age and sex distributions of a starting population (census t1) and an end population (census t2). Ideally, a demographic balance equation should be applied, where the end population = the starting population ± natural increase ± net migration (where natural increase = births—deaths, and net migration = immigrants—emigrants). Figures showing natural increase and net migration were not available. Instead we reconstructed the intercensal population size by cohorts for the ages 10 to 70. Each cohort reconstruction started with the population in census t1. For each cohort the number of deaths from the registers in the intercensal period was subtracted from the population at t1 to estimate the number of survivors at census t2. The difference between the estimated cohort size at t2 and the actual cohort size at t2 was taken to be net migration for that cohort. We assume that net migration equally distributed across the intercensal years.

36
*Norsk Magazin for Lægevidenskaben* 2, 1861, 15, 460–465.

37 Luy, ‘Causes’; Holden, ‘Why Do Women’; Drevenstedt *et al.*, ‘The rise and fall’; Zarulli *et al.*, ‘Women live longer’.

38 Hinde, ‘Sex differentials’.

39 McFarlane, ‘Tuberculosis’.

40 Susan B. Sorenson, ‘Gender Disparities in Injury Mortality: Consistent, Persistent, and Larger Than You'd Think’, *American Journal of Public Health*, 2011, 101, 353–58.

## Notes

**Hilde L. Sommerseth** is Professor of Historical Demography and Director of the Norwegian Historical Data Centre at UiT—the Arctic University of Norway, Norway.

**Evelien C. Walhout** is Assistant Professor in Economic and Social History at Leiden University, The Netherlands.

